# System-Level Offline Time Synchronization Architecture for Distributed Electrical Signal Monitoring Using Raspberry Pi 5

**DOI:** 10.3390/s26082519

**Published:** 2026-04-19

**Authors:** Adriana Burlibaşa, Silviu Epure, Mihai Culea, Cristinel Radu Dache, Cristian Victor Lungu, George-Andrei Marin, Ciprian Vlad

**Affiliations:** 1 Department of Electrical Engineering and Energy Conversion Systems, Faculty of Automation, Computers, Electrical and Electronics Engineering, Dunărea de Jos University of Galati, 800008 Galati, Romania; cristi.dache@ugal.ro (C.R.D.); cristian.lungu@ugal.ro (C.V.L.); george.marin@ugal.ro (G.-A.M.); ciprian.vlad@ugal.ro (C.V.); 2 Department of Electronics, Faculty of Automation, Computers, Electrical and Electronics Engineering, Dunărea de Jos University of Galati, 800008 Galati, Romania; silviu.epure@ugal.ro; 3 Department of Information Technology and Computer Science, Faculty of Automation, Computers, Electrical and Electronics Engineering, Dunărea de Jos University of Galati, 800008 Galati, Romania; mihai.culea@ugal.ro

**Keywords:** Raspberry Pi, offline, synchronized, RTC, multi-sources, PTP, chrony, PHC

## Abstract

Accurate time synchronization is essential in distributed electrical signal monitoring, where phase coherence and event correlation depend on precise timing agreement between acquisition nodes. Conventional approaches often rely on a single synchronization source, typically internet-based Network Time Protocol (NTP) or GPS-disciplined clocks, which is impractical in isolated, offline, or cost-sensitive scenarios. This paper introduces an autonomous offline synchronization architecture for multi-node monitoring systems built on Raspberry Pi 5 (RPI5) platforms connected to a private Ethernet network. Instead of depending on one timing method, the system integrates several complementary mechanisms: battery-backed RTC persistence via the J5 interface, deterministic orchestration through systemd services, automated boot time recovery, chrony-managed NTP discipline, and Precision Time Protocol (PTP) hardware timestamping using PTP Hardware Clock (PHC). Synchronization performance is validated through continuous multi-day measurements of long-term stability, inter-node phase coherence, and short-term jitter. Controlled power-loss scenarios are also included to verify recovery behavior. The system maintains sub-microsecond alignment between nodes using only commodity hardware and no external time source. To further confirm inter-node timestamp alignment at the signal level, both hardware-based reference signal injection and software-based synchronized signal emulation are employed, providing ground-truth validation alongside scalable and reproducible evaluation. The results show that low-cost embedded hardware can support reliable, long-duration synchronization in fully offline installations.

## 1. Introduction

Accurately synchronized monitoring of electrical signals is essential in distributed energy systems, where phase relationship, event timing, and power quality indicators all depend on precise temporal alignment across multiple nodes [1,2,3,4,5,6,7]. Even small timing errors can distort phase estimation, alter frequency calculations, or cause misinterpretation of transient events. This requirement becomes even more challenging in environments that operate offline or in isolation, where access to public networks or an external time reference is intentionally restricted.

Most existing monitoring architectures rely on internet-based Network Time Protocol (NTP) servers available through the internet [8,9,10,11,12,13,14] or on centralized timing hardware with continuous network connectivity [4,15,16,17]. While effective in network infrastructures, these approaches are impractical for secure laboratory environments, remote field installations, and autonomous long-term systems. Alternatives that use dedicated GPS or GNSS timing modules [18,19], or specialized industrial synchronization hardware [15,20,21,22,23], often increase cost and complexity, limiting scalability for low-cost or experimental deployments.

A further limitation of many synchronization strategies is their lack of system-level integration. Some studies examine timing performance under ideal conditions but do not address deterministic startup behavior, automated recovery, or long-term unattended operation [1,15,24]. Work on IEEE 1588 resilience highlights the importance of robustness under faults [25], and industrial evaluations demonstrate sub-microsecond performance in controlled conditions [26,27]. However, these efforts often analyze individual timing mechanisms rather than developing a fully integrated synchronization architecture designed for embedded platforms. Practical acquisition systems must ensure predictable behavior after reboots, reliable clock convergence, and stable restoration following power interruptions [28,29,30]. Manual scripts or ad hoc configuration approaches increase fragility and reduce reproducibility [25].

Several offline synchronization strategies exist for embedded devices, each with trade-offs between complexity and achievable precision [1,31,32,33,34,35]. Many focus on wireless environments, where drift, sampling mismatch, and network latency are major challenges. Approaches such as fractional and adaptive methods in Wireless Body Area Networks (WBANs) systems [36], protocols like Reference Broadcast Synchronization (RBS), Timing-sync Protocol for Sensor Networks (TPSNs), and Flooding Time Synchronization Protocol (FTSP) in sensor networks [37,38,39,40,41,42,43,44] and consensus-based or Kalman filter-based algorithms [45,46] are effective in their respective domains but less suited to wired systems with hardware timestamping capabilities.

Recent improvements in Raspberry Pi 5 (RPI5) make it a strong platform for distributed synchronization tasks [29,30,47,48]. Unlike earlier Raspberry Pi models, which lacked an onboard real-time clock (RTC) and relied on network-based NTP, RPI5 introduces native battery-backed RTC support via the J5 connector [30,47]. Although the Raspberry Pi Zero series also provided an RTC header, its limited networking, typically requiring a USB Ethernet adapter, restricts its suitability for high-precision applications [49].

RPI5 was chosen as the acquisition platform based on a balance of performance, cost, and native features. Industrial-grade devices, such as CompactRIO or Beckhoff embedded PCs, offer robust IEEE 1588 support but are very expensive for multi-node deployments [50,51]. Commodity SBCs like the BeagleBone Black or Jetson boards lack mature PHC support under standard Linux kernels [52], while microcontroller-based platforms such as Arduino or ESP32 cannot simultaneously run PTP daemons, systemd-based orchestration, and a monitoring stack based on Python 3.12.3 [53,54,55]. RPI5 addresses these limitations: it integrates battery-backed RTC functionality [30,48], includes an Ethernet controller (Cadence MACB/GEM) with PHC support exposed via /dev/ptp0 [56,57,58], runs a Debian-based Linux environment with full systemd capability, and remains affordable for three- to five-node experiments [35,59,60].

Raspberry Pi synchronization has typically relied on software-only NTP tools, such as chrony [8,9,12,24]. On RPI5, NTP accuracy is improved by combining it with IEEE 1588 PTP hardware timestamping [27,29,48,61]. LinuxPTP tools like ptp4l and phc2sys allow direct alignment between the PTP Hardware Clock and the system clock, enabling microsecond-level alignment across multiple nodes [25,27]. These capabilities support deterministic boot time restoration, runtime drift mitigation, and coordinated multi-node acquisition.

Unlike previous studies that focus on a single synchronization technique or on wireless, resource-constrained systems, this work presents and experimentally validates a fully integrated offline synchronization architecture for RPI5 acquisition nodes connected through a private wired network. The novelty lies in coordinated exploitation of Ethernet-level PTP hardware timestamping [61,62], driver-level support for the MACB controller [56,57,58], battery-backed RTC persistence via the J5 [30,48], and systemd-based control logic [63] to create a self-contained, automated synchronization system. Systemd service and timer units manage RTC alignment, PHC discipline, network-based convergence, and periodic drift correction during both boot and runtime. The design provides deterministic operation under network isolation, reboots, and both controlled and uncontrolled power cycles. Multi-day experiments demonstrate consistently stable sub-microsecond alignment, long-term drift suppression, and predictable recovery without external time references.

Finally, to validate the synchronization framework, two complementary evaluation approaches are employed: hardware-based validation, which provides a ground-truth reference by injecting a common physical signal via native GPIO pin into all nodes simultaneously, and a software-based validation, which enables controlled, scalable, and repeatable testing of timestamp synchronization behavior under synthetic but precisely aligned conditions. A hardware-based approach allows direct measurement of inter-node timestamp offset, short-term jitter, and long-term drift behavior. Since the input signals are physically identical across all nodes, any observed discrepancies can be attributed solely to synchronization and acquisition timing effects. For a software-based approach, a customizable monitoring dashboard was implemented using Plotly (v6.4.0) visualization library and the Dash (v3.3.0) web framework [64]. This provides synchronized multi-panel visualization of time-aligned signals and supports offline analysis through NumPy, SciPy, and related libraries [64,65]. Combined with inexpensive hardware and open-source software, the proposed approach offers a practical and reproducible platform for distributed monitoring in scenarios where traditional network-dependent synchronization methods are non-viable. Together, these approaches enable validation of both the physical synchronization accuracy and the system-level behavior of timestamped data acquisition.

The rest of this paper is organized as follows. Section 2 presents the system architecture and network topology. Section 3 details the clock control mechanism, including RTC management, chrony-based synchronization, and PHC-to-system discipline, as well as long-duration experimental validation and performance evaluation through statistical analysis and key performance indicators (KPIs). Section 4 validates timestamp synchronization at the signal acquisition level, both experimentally and through a fully controlled simulation framework. Section 5 discusses system-level implications, robustness considerations, and future research directions. Section 6 concludes the paper.

## 2. Hardware Configuration and Network Offline Management

**Figure 1:**
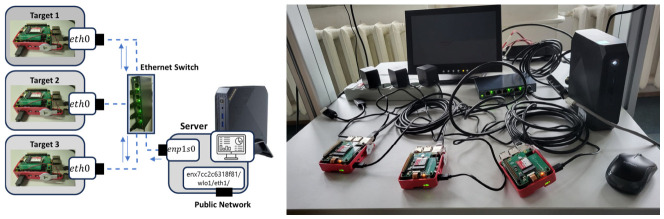
Experimental setup for a distributed-system architecture.

The experimental setup consists of four machines: one server node and three RPI5 target nodes (see Figure 1). The server is an Intel NUC system that provides internet connectivity and administrative access via secure shell (SSH) or remote desktop. In addition to its external network interface, the server is equipped with a dedicated Ethernet interface configured within a private local network used exclusively for communication with the RPI5 targets.

The monitoring dashboard, implemented using the Plotly Dash framework, is hosted on the server node. Due to its dual-network configuration, the dashboard can be accessed by authorized users via IP address or hostname, while the RPI5 targets remain isolated from direct internet connectivity during experimental operation. The three RPI5 devices operate within the private network and are configured in a master–client topology, consisting of one master clock node and two client clock nodes, as will be described in a following section.

All RPI5 targets run a Debian-based Linux distribution (kernel version 6.12.47+rpt-rpi-2712, aarch64), with native support for real-time clock (RTC) functionality. Although the platform normally permits synchronization with online time sources [7,18,23], the RPI5 nodes are intentionally configured to operate fully offline, without access to external NTP servers.

The server node is the only system synchronized to an internet-based NTP service [13,14] and serves exclusively as a reference for initial configuration and maintenance. During the setup phase, temporary routing and access rules are enabled on the server to allow package installation and dependency updates on the RPI5 targets. A static IP address must be assigned to the server on the network PCI (e.g., enp1s0) connected to the RPI5 devices, ensuring stable addressing within the isolated environment. IP forwarding is enabled, sudo sysctl -w net.ipv4.ip_forward=1, to allow controlled routing between its external network interface and the private network during system setup. To enable initial software installation on the RPI5 targets, the server temporarily provides controlled external network access by applying network address translation (NAT) rules on the private interface.

sudo iptables -A FORWARD -i enp1s0 -o enx7cc2c6318f81 -j ACCEPT sudo iptables -A FORWARD -i enx7cc2c6318f81 -o enp1s0 -m state --state RELATED, ESTABLISHED -j ACCEPT
sudo iptables -t nat -A POSTROUTING -o enx7cc2c6318f81 -j MASQUERADE”

After system configuration is finalized, these permissions are revoked by removing the NAT rule: sudo iptables -t nat -D POSTROUTING 1. The interface name enx7cc2c6318f81 corresponds to the network adapter through which the server node accesses the public network. Depending on the available hardware, this external interface may instead be a native Ethernet device (e.g., eth0, eno1) or a wireless interface (e.g., wlo1). Consequently, the network configuration commands must be adapted to match the actual external network interface present on the server system [63]. On each RPI5 target, the following configuration is applied to/etc/systemd/network/10-eth0.network:

[Match]
Name=eth0
 
[Network]
Address=<RPI5_IP>/24
Gateway=<server_IP_LocalNetwork>
DNS=8.8.8.8
 
sudo systemctl enable systemd-networkd
sudo systemctl restart systemd-networkd
 
sudo bash -c ‘echo “nameserver 8.8.8.8” > /etc/resolv.conf’
sudo bash -c ‘echo “nameserver 1.1.1.1” >> /etc/resolv.conf’
sudo chattr +i /etc/resolv.conf
sudo systemctl restart systemd-networkd

The correct application of the network configuration is verified using networkctl status eth0, which confirms that the corresponding system-networkd configuration file (e.g., /etc/systemd/network/10-eth0.network) has been loaded at the network interface level and that the interface is in a routable and configured state. Proper operation of the network stack further requires that the systemd-networkd service is active. To avoid conflicts in network interface management, it is also verified that NetworkManager is not running. If active, the service is stopped and disabled [63]. Running both systemd-networkd and NetworkManager concurrently can lead to contention over network interfaces, resulting in unstable behavior, such as intermittent connectivity, interface state flapping, or DHCP failures, as documented in standard Linux system service management references [63].

The server system time can be initialized either manually using the system date utility or automatically via a standard NTP-based synchronization mechanism [12,13]. To ensure a universal and unambiguous time reference across all nodes, Coordinate Universal Time (UTC) is used consistently for both the system clock and the RTC on the server and all RPI5 targets [32]. Each RPI5 device is equipped with an external lithium-ion backup battery (UN 3090), connected to the J5 connector, enabling RTC time preservation across power interruptions [30]. To enable correct battery detection and RTC operation, specific configuration directives are applied in the boot firmware configuration file (/boot/firmware/config.txt), including the activation of RTC support and the removal or deactivation of conflicting default settings. /boot/firmware/config.txt| grep -i dt:

dtparam=i2c_arm=on
#dtparam=i2s=on
#dtparam=spi=on
dtparam=audio=on
dtoverlay=vc4-kms-v3d
dtparam=rtc_enable=on
#dtparam=rtc=bbat_vchg=3000000
#dtoverlay=dwc2,dr_mode=host

After applying the firmware configuration changes and rebooting the target system, the RTC device (rtc0) becomes available and operational. Correct initialization is verified through kernel log messages and system device listings, which confirm successful registration on the RTC driver during system startup.

[ 0.252682] rpi-rtc soc@107c000000:rpi_rtc: registered as rtc0
[ 0.254050] rpi-rtc soc@107c000000:rpi_rtc: setting system clock to 1970-02-22T05:12:48 UTC (4511568)

The presence of the RTC device is further confirmed by the corresponding sysfs entry: lrwxrwxrwx 1 root root 0 Feb 22 1970 /sys/class/rtc/rtc0 -> ../../devices/platform/soc@107c000000/soc@107c000000: rpi_rtc/rtc/rtc0

Care must be taken when configuring RTC-related firmware parameters. In particular, the directive dtparam=rtc=bbat_vchg=3000000 must not be enabled when using a lithium-ion backup battery connected to the J5 connector of RPI5. Although this parameter is frequently recommended in online documentation, it applies only to RTC backup batteries connected via the I^2^C interface and is not compatible with the J5-based RTC configuration [48]. When enabled in this context, the parameter prevents successful initialization of the rtc0 device.

When a new J5 backup battery is installed, the RTC typically contains a default reference timestamp set far in the past. As a result, reading date (e.g., via /sys/class/rtc/rtc0/date) initially returns a value such as 1970-02-22 or 1970-04-06. This behavior is expected and reflects the absence of a previously stored valid timestamp. The RTC can be initialized to the correct UTC time using the dedicated hwclock utility. The hwclock binary is available by default on some Linux distributions; in the present setup, it was installed via the Debian package util-linux-extra. After verifying the presence of the binary (e.g., /sbin/hwclock), the RTC time is updated by writing the current system time to the J5 backup battery using the command hwclock -w. For example, if the RTC initially reports 1970-02-24 05:01:47.005359+00:00 and the system time is Mon 15 Dec 16:41:24 UTC 2025, executing hwclock -w updates the RTC to 2025-12-15 16:41:51.060779+00:00.

The RTC initialization is a one-time, persistent operation. Once completed successfully, the updated RTC value is confirmed during system boot through kernel log messages, such as: rpi-rtc soc@107c000000:rpi_rtc: setting system clock to 2025-12-15T16:41:51 UTC (1765452364). It should be noted that hwclock operates strictly in UTC. Therefore, if the system clock is configured to use the local time zone, a constant offset will appear between the system time and the RTC time, which may be misinterpreted as clock drift. For this reason, both the system clock and the RTC are configured to operate exclusively in UTC throughout this work.

## 3. System and RTC Clock Control

The objective of this section is to detail the layered clock control architecture that enables fully autonomous time alignment in an offline, multi-node setting.

The operative scenario imposes three key constraints: no access to external NTP servers or GPS references at runtime, so all-time discipline must be derived internally; nodes are subject to unscheduled power interruptions, requiring deterministic restoration of a valid time reference at every boot; and inter-node alignment must remain bounded to sub-millisecond levels for chrony-based deployments, and sub-microsecond levels when PTP hardware timestamping is enabled, to support correct phase relationship estimation in 50 Hz electrical signal acquisition.

To satisfy these constraints simultaneously, the architecture is structured as three cooperating layers: (1) RTC-to-system restoration at boot time, ensuring temporal continuity across power cycles; (2) network-based synchronization via chrony or PTP, progressively disciplining the system clock during runtime; and (3) periodic system-to-RTC write-back, preventing long-term RTC drift from undermining the boot time restoration guarantee.

The following subsections describe the configuration, implementation, and validation of each layer.

### 3.1. Clock Synchronization Between Target Nodes

**Figure 2:**
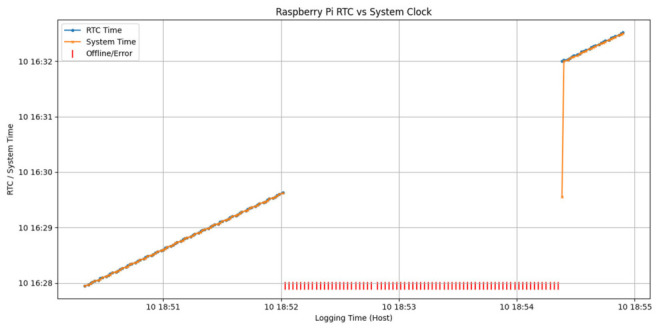
RTC time (blue) and system clock time (orange), systemd service correction.

At startup, the system clock is initialized from the RTC, which is maintained by a backup battery. As expected, during power-off intervals, the system clock halts while the RTC continues to advance, resulting in an offset after reboot. To ensure temporal continuity in offline operation, the system clock is restored from the RTC at boot time using a systemd service (see Figure 2):

 
echo “Creating rtc-to-system.service”
sudo tee /etc/systemd/system/rtc-to-system.service > /dev/null <<‘EOF’
[Unit]
Description=Sync system clock from RTC at boot
After=multi-user.target
 
[Service]
Type=oneshot
ExecStart=/bin/bash -c ‘date -s “$(cat /sys/class/rtc/rtc0/date) $(cat /sys/class/rtc/rtc0/time)”’
RemainAfterExit=yes
 
[Install]
WantedBy=multi-user.target
EOF

The service executes automatically during system startup and aligns the system clock with the RTC before any time-dependent services are launched. As illustrated in Figure 2, the system-based mechanism ensures immediate alignment between the system time and the RTC at boot, effectively eliminating the offset observed after power loss and enabling fully unattended operation in offline environments.

The analysis is further extended to a two-target scenario; therefore, the described configuration is applied identically to a second target. Both targets operate in offline mode. The objective of the next experiment is to evaluate the relative drift between the system clocks and the RTC clocks of the two targets during prolonged autonomous operation. To ensure that any observed drift is not influenced by the initialization procedure, time samples are collected simultaneously from both targets. System time values are retrieved in UTC with millisecond resolution using the command date -u +’%Y-%m-%d %H:%M:%S.%3N’, while RTC time values are read directly from the rct0 device (/sys/class/rtc/rtc0/time) on each target. All measurements are acquired in parallel to minimize sampling-induced offsets.

As it is shown in Table 1, at the beginning of the experiment, the system clocks and RTC clocks of both targets are fully aligned.

**Figure 3:**
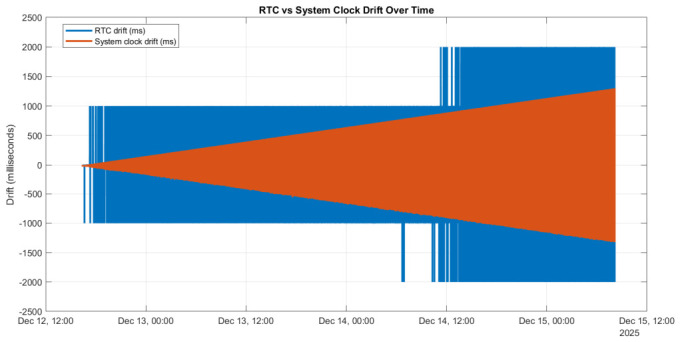
RTC vs. system clock drifts over time for two RPI5.

After more than 48 h of continuous offline operation, relative drifts of approximately 1–2 s were observed between the timestamps reported by the two devices. Figure 3 illustrates the temporal evolution of these drifts. Both the system clock drift and the RTC drift increase over time, but with distinct characteristics.

The system clock drift exhibits a near-linear trend, whereas the RTC drift evolves in discrete steps, reflecting the independent oscillators and limited resolution of the RTC hardware. For electrical signal monitoring applications, particularly those involving 50 Hz signals and millisecond-scale acquisition periods, such drift levels are unacceptable. Consequently, additional synchronization mechanisms are required to maintain long-term time alignment between the two targets. To address this limitation, two offline synchronization strategies are investigated in the following sections: an NTP-based master-client architecture implemented using chrony and a PTP-based solution implemented either through chrony-assisted synchronization or direct physical hardware clock (PHC)-to-system clock alignment. All approaches are evaluated using the same two-target setup to enable a direct comparison of achievable synchronization accuracy and system complexity.

#### 3.1.1. NTP Master–Client Synchronization Using Chrony

**Figure 4:**
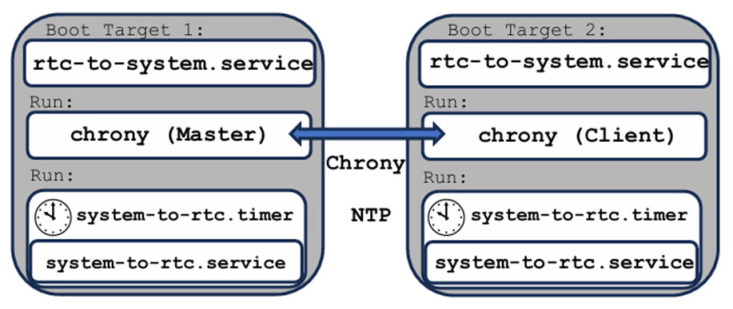
Synchronization architecture combining RTC-to-system initialization, chrony-based NTP synchronization, and periodic system-to-RTC correction.

The synchronization architecture based on NTP using chrony is illustrated in Figure 4. This solution targets millisecond-level time synchronization and is suitable for multi-source electrical signal measurement systems where strict sub-microsecond alignment is not required.

Chrony [66,67] is a flexible and robust implementation of the NTP capable of synchronizing the system clock using external NTP servers, reference clocks, or peer nodes while also providing NTP services to other systems on the network. In the proposed offline architecture, crony is used exclusively in a master–client configuration, where one RPI5 target operates as the NTP master and the second target synchronizes its system clock to this reference. Chrony is not enabled by default on standard Linux distributions and must be explicitly installed. Upon installation, the default systemd-timesyncd service is removed, as chrony and systemd-timesyncd are mutually exclusive time synchronization services. Linux distributions are designed to allow only one service to control the system clock at a given time to prevent conflicts [66,67,68]. In the two-target setup, one RPI5 node is configured as the chrony master, while the second node operates as a chrony client. The corresponding chrony configuration files (/etc/chrony/chrony.conf) for the master and client roles are summarized in Table 2.

After applying the configuration changes and enabling the chrony service, synchronization status is verified using standard system tools. On the client node, the timedatectl utility confirms synchronization through the status System clock synchronized: yes. Additionally, the chronyc sources command confirms the active master reference, as illustrated below:

 
MS Name/IP address  Stratum Poll Reach LastRx Last sample
===================================================
^* <RPI5_masterIP> 8 6 377  19   -1456ns[-2028ns] +/-     94us\
 

**Figure 5:**
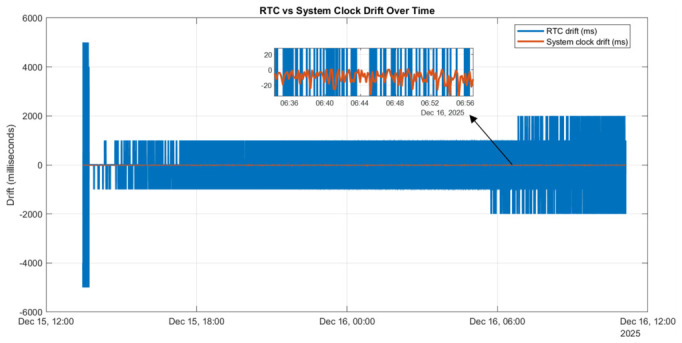
Evolution of RTC and system clock drift over time with chrony-based synchronization.

These measurements indicate that the system clock drift between the two targets is effectively constrained within the expected millisecond-level range. However, while chrony successfully stabilizes the system clocks, it does not fully eliminate drift between the RTC backup batteries on the two devices, as shown in Figure 5.

Chrony provides the rtcsync directive [66], which periodically updates the RTC from the system clock. In theory, this mechanism should reduce RTC drift; however, experimental results show that RTC-to-RTC drift persists over extended offline operation when relying on rtcsync alone. To further mitigate RTC drift, the RTCs on both the master and client nodes were manually aligned using the hwclock -w operation. This procedure significantly reduced the observed RTC drift and demonstrated that periodic RTC correction is effective. However, applying this correction too frequently introduces minor temporal perturbations due to the write operation itself. In this paper, a cadence of one hour was selected as a compromise between drift reduction and system stability, although longer intervals (e.g., 5–6 h) are also feasible. To automate this process and eliminate manual intervention, a dedicated system timer–service pair was introduced to periodically synchronize the RTC with the system clock. The timer triggers a one-shot service that updates the RTC and then terminates immediately, ensuring minimal runtime overhead.

sudo tee /etc/systemd/system/system-to-rtc.service > /dev/null <<‘EOF’
[Unit]
Description=Sync RTC from system clock
After=time-sync.target chrony.service
Wants=time-sync.target
 
[Service]
Type=oneshot
ExecStart=/sbin/hwclock --systohc --utc
EOF
 
sudo tee /etc/systemd/system/system-to-rtc.timer > /dev/null <<‘EOF’
[Unit]
Description=Periodic system clock to RTC sync
 
[Timer]
OnBootSec=5min
OnUnitActiveSec=1h
AccuracySec=1min
Persistent=true
 
[Install]
WantedBy=timers.target
EOF

The output status listed confirms that the system-to-rtc.timer unit is enabled and actively waiting, while the corresponding system-to-rtc.service executes successfully and transitions to an inactive (dead) state after completion.

**Figure 6:**
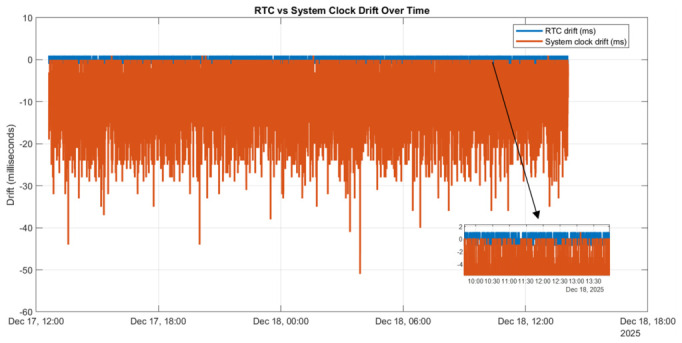
RTC and system clock drift after introducing periodic system-to-RTC correction.

This behavior is expected and confirms correct periodic RTC maintenance without continuous resource consumption. Long-term measurements indicate that, with this mechanism in place, both the system clock drift and the RTC drift remain bounded within a few milliseconds (see Figure 6).

**Figure 7:**
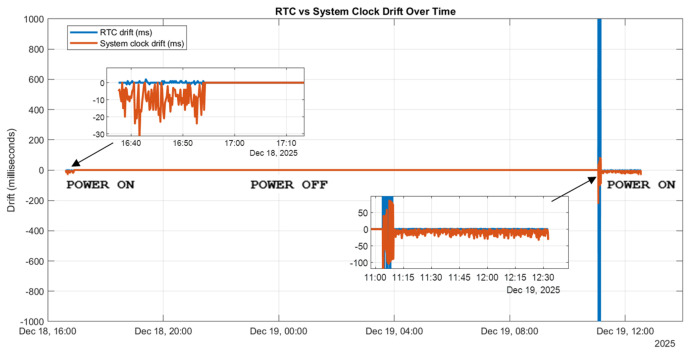
Drift behavior across power-off and power-on cycles using chrony-based synchronization.

This behavior represents a significant improvement over the unmanaged offline case and is well within acceptable limits for millisecond-scale electrical signal acquisition. To assess whether this configuration meets production-grade requirements for offline operation, additional experiments were conducted across power-off and power-on cycles. Figure 7 illustrates the drift behavior when both RPI5 targets are powered down simultaneously and then restarted.

Immediately after reboot, a slightly increased offset is observed; however, the system stabilizes rapidly, and the drift converges to the expected milliseconds range within a short period. This behavior demonstrates that the proposed chrony-based architecture provides reliable time continuity during runtime and acceptable convergence after power interruptions.

Overall, this setup offers a robust millisecond-accuracy solution for offline synchronized monitoring. Beyond passive measurement applications, the achieved synchronization accuracy is sufficient to support coordinated actions, scheduled tasks, and distributed operations that depend on a shared time reference across multiple targets.

#### 3.1.2. PTP-Based Synchronization Using Chrony and the PHC

**Figure 8:**
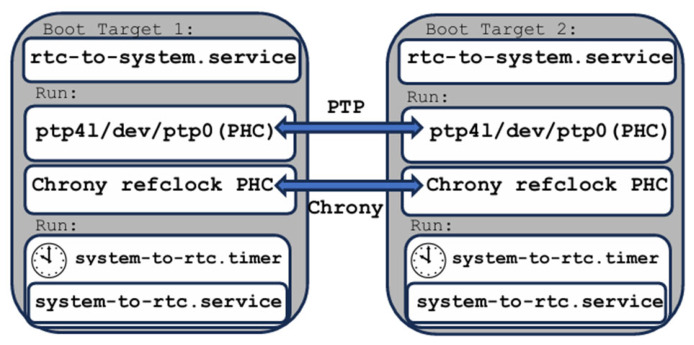
Synchronization architecture combining RTC-to-system initialization, PTP-based PHC synchronization, chrony-based system clock discipline, and periodic system-to-RTC correction.

To achieve synchronization accuracy beyond the millisecond level provided by NTP, a Precision Time Protocol (PTP)-based solution was evaluated (see Figure 8). PTP enables microsecond- and sub-microsecond-level synchronization when supported by hardware timestamping and is, therefore, well suited for high-resolution electrical signal monitoring in offline environments [24,27].

Both RPI5 targets are equipped with Ethernet controllers based on Cadence MACB/GEM architecture, driven by the macb kernel driver [56,57,58] under Linux kernel version 6.12.47+rpt-rpi-2712. This Ethernet MAC provides hardware timestamping capabilities and exposes a PTP Hardware Clock (PHC), visible through the /dev/ptp0 device node. The presence of this device confirms that the platform supports hardware-assisted PTP synchronization [61]. Hardware timestamping support can also be verified with the command ethtool -T <RPI5_ethInterfaceName>. The reported capabilities include hardware transmit timestamping, hardware receive timestamping, and the presence of a PHC, confirming that the network interface can support PTP at the MAC level.

In the earlier NTP-based configuration, three system services were employed to ensure millisecond-level clock accuracy at boot time, during runtime, and across power-on/off cycles. When transitioning to a PTP-based solution, the RTC-to-system and system-to-RTC services remain applicable and continue to ensure time continuity during power cycling. However, the chrony configuration must be adapted to integrate with PTP.

Chrony is responsible for disciplining the system clock (CLOCK_REALTIME) and supports multiple time sources, including NTP servers and PTP via a PHC [66,68]. A key aspect of this setup is that chrony does not automatically select PTP as a synchronization source; explicit configuration is required to ensure that the PHC is used as the reference clock. In the proposed architecture, PTP is employed to synchronize the PHC of each device using hardware timestamping, while chrony disciplines the system clock based on the PTP-synchronized PHC rather than external NTP servers. This separation of responsibilities ensures that high-precision synchronization is achieved at the hardware level through PTP, while chrony provides system-level time stability and propagates the synchronized time to the RTC.

Under Linux, PTP support is divided between kernel and user space and is implemented through the linuxptp framework, which provides a standard-compliant IEEE 1588v2 implementation [61]. This framework includes the ptp4l daemon, which synchronizes the PHC to a PTP grandmaster, and the phc2sys utility, which can synchronize the system clock directly from the PHC when hardware timestamping is enabled [61]. In the present architecture, however, direct PHC-to-system synchronization via phc2sys is not yet applied, as the system clock is disciplined by chrony using the PHC as a reference source. The use of phc2sys and its role in alternative synchronization configurations are addressed in a subsequent section.

For initial validation, the ptp4l daemon can be launched manually on both master and client RPI5 targets. On the master node, ptp4l is started in grandmaster mode (/usr/sbin/ptp4l -i eth0 -H -2 -m), while on the client node, it is started in slave mode (/usr/sbin/ptp4l -i eth0 -H -2 -s -m). In both cases, standard output and error streams are redirected to a dedicated log file for monitoring and diagnostic purposes. To prevent permission-related startup failures, the log file must exist prior to launching ptp4l and have appropriate access rights. In our implementation, this was ensured on both targets by creating the log file and setting read permissions accordingly before executing the ptp4l commands.

When the daemon is started successfully, the generated log files on the master and client nodes contain characteristics, state transitions, and synchronization messages that confirm correct PTP operation. These log traces (e.g., /var/log/ptp4l.log) provide clear evidence on the master node. PHC selection: selected /dev/ptp0 as PTP clock; port initialization: INITIALIZING to LISTENING on INIT_COMPLETE; and role assignment and grandmaster election: selected local clock 2ccf67.fffe.f62eb3 as best master, ptp4l[6798.380]: port 1 (eth0): assuming the grand master role

The client node detects the remote grandmaster and disciplines its PHC accordingly. Evidence described in the log is PHC detection: selected /dev/ptp0 as PTP clock; remote master discovery: new foreign master 2ccf67.fffe.f62eb3-1, best master selection: selected best master clock 2ccf67.fffe.f62eb3; state transitions: LISTENING to UNCALIBRATED on RS_SLAVE, UNCALIBRATED to SLAVE on MASTER_CLOCK_SELECTED; and continuous offset reporting:

ptp4l[9002.939]: master offse -67 s2 freq -6796 path delay 2748
ptp4l[9003.939]: master offset 7 s2 freq  -6742 path delay 2729

Rather than manually verifying whether the ptp4l process is running correctly, long-term and unattended operation can be achieved by deploying dedicated systemd service units. These services automate the activation of ptp4l at boot time and provide continuous supervision, enabling automatic restart and failure tracking during extended operation. The corresponding service unit definition used in this work is summarized in Table 3.

When ptp4l is managed through a system service unit, pre-creating the log file is no longer required, as it is automatically created when the service is enabled and started. For long-running deployments without human intervention, log file growth must be controlled to prevent excessive disk usage [61,63].

This is addressed by configuring log rotation through a dedicated logrotate rule located in /etc/logrotate.d/ptp4l. The following configuration was applied:

/var/log/ptp4l.log {
    daily
    rotate 7
    missingok
    notifempty
    copytruncate}

The configuration rotates the log file daily and retains the seven most recent rotations, automatically removing older entries. The missingok directive prevents an error if the log file does not yet exist, while notifempty avoids rotating empty log files. The copytruncate option allows the ptp4l process to continue writing to the same file description without requiring a service restart, making this approach suitable for continuous, unattended operation.

The effectiveness of log rotation was confirmed experimentally. On a system without log rotation enabled, the ptp4l log file grew to approximately 86 MB over time, whereas on a system with log rotation enabled, the log file size remained limited to approximately 1.5 MB. This demonstrates that the proposed logging strategy effectively bounds disk usage and prevents resource exhaustion in long-term deployments.

The ptp4l daemon disciplines only the PHC and does not directly modify the system clock. System synchronization is, therefore, achieved indirectly through chrony, which must be explicitly configured to discipline the system clock (CLOCK_REALTIME) using the PHC as its reference. To enable this behavior, the chrony configuration is modified to include the PHC as a reference clock. The directive refclock PHC /dev/ptp0 instructs chrony to synchronize the system clock from the PHC, while the parameters poll 0 and dpoll -2 enable fast, low-latency updates, which are well suited for local area PTP deployments. With this configuration, the system clock is effectively locked to the PTP-disciplined PHC and no longer derives time from external NTP sources.

Table 4 summarizes the required /etc/chrony/chrony.conf settings for both the master and client targets. The directive local stratum 8 allows downstream clients to synchronize if required. It is important to emphasize that on the master node, the PHC is disciplined by ptp4l and, therefore, serves as the primary time source. In this configuration, chrony does not independently obtain time from external servers; instead, it tracks the PHC as a reference clock. The use of local stratum 8 does not override the PHC as the reference source, and the PHC remains a stratum 1 time source internally.

After applying the configuration changes, a daemon-reload was performed, and the chrony service was enabled and started to ensure persistent operation. At startup, chronyd (v4.6.1) loaded the stored frequency calibration parameters from /var/lib/chrony/chrony.drift, initially reporting a frequency estimate of 0.000 +/- 1000000.000 ppm prior to convergence. Shortly thereafter, chrony selected PHC0 as the active reference source, as confirmed by the service logs: Started chrony.service - chrony, an NTP client/server. Selected source PHC0. This confirms that the system clock (CLOCK_REALTIME) is disciplined directly from the PHC, rather than from external NTP servers.

Verification using the chronyc sources command further confirms that the PHC0 is selected as the active synchronization source, with measured offsets on the order of tens of microseconds.

 
MS Name/IP address Stratum Poll Reach LastRx Last sample
==============================================================
#* PHC0        0  0 377  0  +30ns[ +42ns] +/-  55ns
 

Further validation using the chronyc tracking command shows that the PHC is selected as the reference source with Stratum: 1. The reported system time deviation, for example, System time: 0.000000012 s fast of NTP time and a Root Mean Square (RMS) offset of 39 ns, demonstrated high-precision time synchronization of the system clock relative to the PTP-disciplined PHC.

It is important to distinguish this behavior from that of the hardware real-time clock (RTC). In Linux-based systems, the RTC is not designed for high-precision timekeeping [63]. Typical RTC devices operate with a 1 Hz tick rate and expose time information only at second-level resolution (e.g., via /sys/class/rtc/rtc0/time) [48]. Even when periodically disciplined, RTC updates occur infrequently and are intended solely to correct long-term drift rather than to maintain real-time accuracy. As a result, nanosecond- or microsecond-level precision cannot be achieved on RTC, regardless of the accuracy of the underlying PTP synchronization. In a correctly functioning system, short-term time stability and high-precision synchronization are provided by the system clock, which can be maintained within a microsecond or better accuracy, while the RTC serves as a coarse long-term reference. Drift on the order of milliseconds to seconds over extended periods is, therefore, normal and expected for RTC hardware and does not indicate a malfunction of the PTP or system-level synchronization mechanism.

To obtain meaningful results, drift analysis must be performed using the timing metrics reported directly by the synchronization services, specifically by tracking the master offset values from ptp4l and the frequency and offset estimates provided by chrony over time (e.g., via chrony traking).

**Figure 9:**
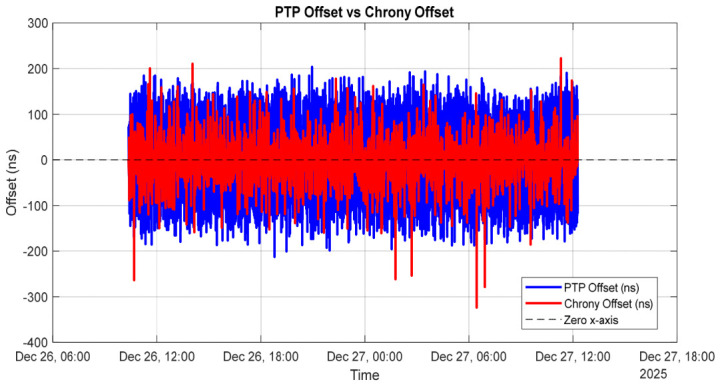
Comparison between PTP master–slave offset and chrony system clock offset.

Figure 9 presents a direct comparison between the clock offset reported by the PTP servo (ptp4l) and the system clock offset reported by chrony during steady-state synchronized operation.

The PTP offset (blue trace) represents the master–client time deviation measured at the hardware level between the PHCs, while the chrony offset (red trace) reflects the system clock deviation relative to the PTP-disciplined PHC. Both offsets remain centered around zero, with fluctuations confined within a few hundred nanoseconds. The slightly larger variability observed in the chrony offset is expected, as chrony disciplines a software-based system clock that is subject to operating system scheduling effects and filtering dynamics, whereas ptp4l operates directly on the hardware clock. The absence of drift, bias, or long-term trends in either trace confirms stable servo operation and demonstrates that chrony accurately tracks the PTP-disciplined PHC without introducing additional instability. The close alignment between the two traces validates the effectiveness of the combined PTP and chrony architecture and confirms nanosecond-level synchronization between hardware and system clocks.

**Figure 10:**
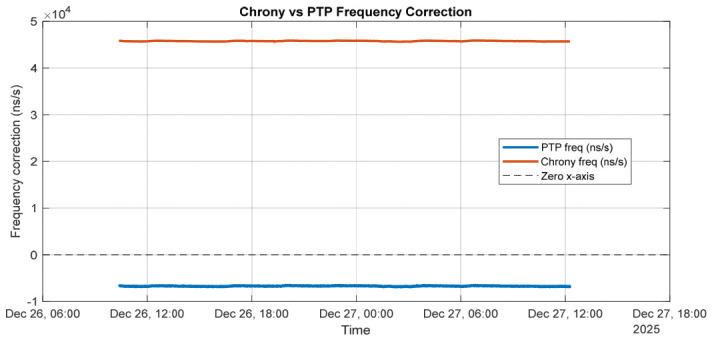
Frequency corrections applied by the PTP servo and by chrony.

Chrony closely tracks the PTP-disciplined PHC, applying smooth and stable frequency corrections through its internal filtering mechanisms. Figure 10 illustrates the frequency corrections applied by the PTP servo and by chrony over the measurement interval. The PTP frequency correction represents the adjustment applied to the hardware PHC oscillator to discipline it to the PTP grandmaster, whereas the chrony frequency correction corresponds to the adjustment applied to the system clock relative to the PTP-disciplined PHC.

Although the magnitudes of the two corrections differ substantially, this behavior is expected, as the servos regulate different oscillators with distinct intrinsic frequency characteristics. The PHC requires a relatively small and stable correction (approximately −6 ppm), indicating good oscillator quality, while the system clock exhibits a large but steady correction (approximately +45 ppm), which is typical of a less accurate crystal oscillator.

The absence of large fluctuations or long-term drift in either trace demonstrates stable servo behavior and confirms that chrony effectively tracks the PTP-disciplined PHC without introducing additional instability into the system clock.

**Figure 11:**
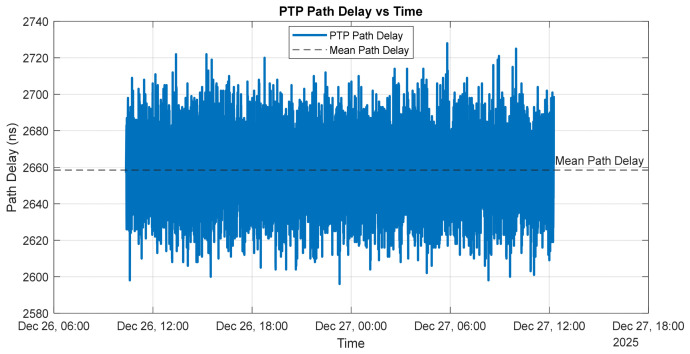
PTP path delay between the grandmaster and the slave.

Figure 11 illustrates the estimated PTP path delay between the grandmaster and the slave over the measurement interval.

The path delay remains centered around a mean value of approximately 2.65 µs, with short-term fluctuations on the order of several tens of nanoseconds. This behavior indicates a stable network topology with no evidence of long-term drift or step changes in propagation delay. The observed high-frequency variations are primarily attributable to timestamping noise, switch queuing effects, and minor packet delay variations inherent to Ethernet-based communication, rather than to changes in the physical path length. The absence of abrupt discontinuities or sustained trends confirms that the network conditions are stable and suitable for high-accuracy PTP time synchronization.

#### 3.1.3. PTP-Based Synchronization Using phc2sys and the PHC

**Figure 12:**
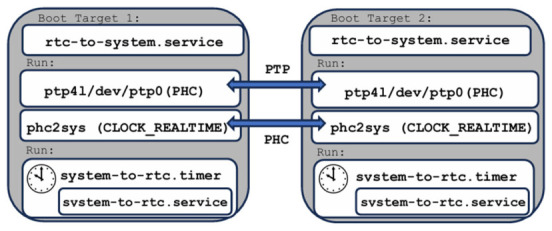
Synchronization architecture combining RTC-to-system initialization, PTP-based PHC synchronization, phc2sys system clock discipline, and periodic system-to-RTC correction.

As an alternative to the chrony-based approach, system clock synchronization can be achieved using phc2sys (see Figure 12), which represents the classic synchronization architecture provided by the linuxptp framework [61]. In this configuration, the system clock is disciplined directly from the PHC, eliminating chrony from the time-disciplining path. To transition cleanly to a phc2sys-based setup, the chrony service is stopped and disabled. Following this step, the timedatectl command confirms that network time synchronization is inactive (e.g, NTP service: inactive), reflecting the removal of chrony from system time management.

Similar to ptp4l, the phc2sys binary may be started manually for validation purposes. Prior to execution, a log file /var/log/phc2sys.log with appropriate permissions can be created to avoid permission-related issues. The phc2sys process is then launched: /usr/sbin/phc2sys -s /dev/ptp0 -c CLOCK_REALTIME -O 0 -m.

In this invocation, the -s /dev/ptp0 option specifies the PHC as the source clock, while -c CLOCK_REALTIME identifies the system clock as the target to be disciplined. The -O 0 parameter applies no fixed offset, ensuring direct alignment between the two clocks, and the -m option enables runtime logging to facilitate observation and verification of synchronization behavior during operation.

CLOCK_REALTIME phc offset  -12 s2 freq +40477 delay 55
CLOCK_REALTIME phc offset -1 s2 freq +40485 delay 55

If phc2sys is executed in the background without explicit log redirection, its runtime output can be accessed via the system journal.

For unattended and long-term operation, manual execution is replaced by system service units that automatically start phc2sys at boot time (see Table 5). To deploy the service-based configuration, any previously running instances of the phc2sys binary must first be terminated, and manually created log files must be removed to avoid conflicts. The services are then enabled and started on both master and client nodes. After activation, log outputs comparable to those obtained during manual execution are expected either in /var/log/phc2sys.log or via journalctl, confirming correct operation. For long-term, unattended deployments, log management must be implemented to prevent uncontrolled disk usage. Similar to the ptp4l configuration, the log rotation for phc2sys is defined in /etc/logrotate.d/phc2sys with the same policy as in the case of ptp4l. For quantitative analysis, offset values were extracted directly from the synchronization logs over a defined measurement interval, “master offset”, from the ptp4l log, and “phc offset”, from the phc2sys log.

**Figure 13:**
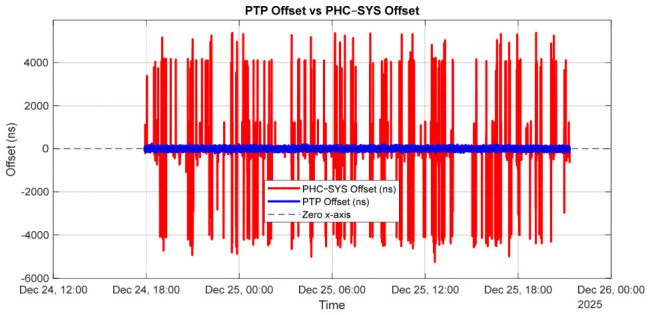
Comparison between PTP master–slave offset and phc2sys offset.

The results presented in Figure 13 reveal a fundamental difference in behavior between chrony-based and phc2sys-based system clock discipline. In relation to Figure 9, Figure 13 shows that chrony applies a smooth and conservative control strategy, using gradual frequency adjustments to effectively filter measurement noise and suppresses short-term jitter.

**Figure 14:**
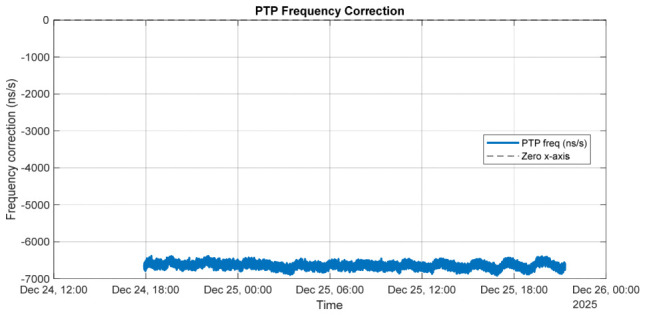
Frequency corrections applied by the PTP servo.

**Figure 15:**
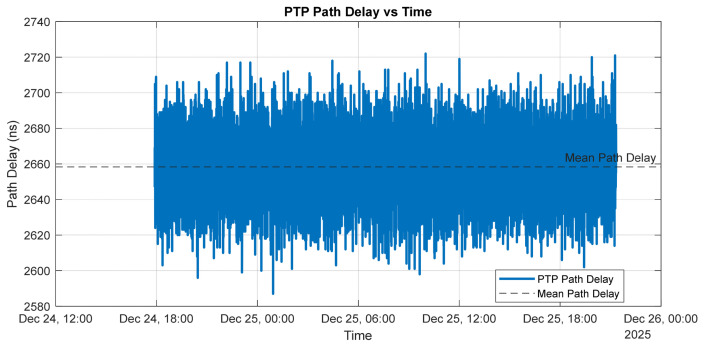
PTP path delay between the grandmaster and the slave.

According to Figure 14 and Figure 15, this approach enhances long-term frequency stability and robustness in environments affected by network asymmetry, packet delay variation, or mixed synchronization sources. In contrast, phc2sys applies faster and more direct corrections, achieving tighter phase alignment and reduced latency, but with less aggressive noise filtering. When hardware timestamping is stable and reliable, phc2sys provides superior short-term precision and smaller time offsets.

Chrony is, therefore, preferable in deployments with oscillator instability, software timestamping, PPS availability, or strict phase alignment requirements. It is particularly appropriate for applications demanding nanosecond-level synchronization accuracy.

### 3.2. Quantitative Comparison Between Chrony and phc2sys

A controlled comparative experimental study was conducted to evaluate the performance of chrony-based and phsc2sys-based system clock discipline under identical operating conditions. Each configuration was assessed for over 24 h independently to isolate its specific impact on system clock synchronization behavior. Short-duration measurements may underestimate instability and fail to capture rare but significant excursions.

chrony-based configuration: In the first configuration, chrony was enabled on the client node as the system clock discipline mechanism, while phc2sys was disabled. The system clock (CLOCK_REALTIME) was synchronized indirectly using the PHC as its reference source. The time offset between the PHC and the system clock was measured using the phc_ctl utility in comparison mode: sudo phc_ctl /dev/ptp0 cmp. This command reports the instantaneous PHC–system clock offset: phc_ctl[512877.977]: offset from CLOCK_REALTIME is 214ns. Offset samples were collected at ten-second intervals over a 24 h observation window and logged to a CSV file for offline analysis. This configuration allows characterization of offset distribution, short-term jitter, and the occurrence of outliers under chrony’s filtering and control strategy.

phc2sys-based configuration: In the second configuration, chrony was disabled and phc2sys was enabled, allowing the system clock to be disciplined directly by the PHC. The same PHC–system clock offset measurement method was applied, using identical sampling intervals and observation duration. This ensured full comparability between the two discipline mechanisms.

For both configurations, key performance indicators were derived from the recorded PHC–system clock offset time series (see Table 6). These included the mean offset, RMS offset, standard deviation, and maximum and minimum observed offsets. Together, these metrics quantify steady-state accuracy, short-term stability, and extreme deviations in clock alignment. The mean time offset observed with phc2sys is approximately 50 ns, indicating reasonably good centering of the system clock relative to the PHC. In comparison, chrony exhibits a higher mean offset of approximately 129 ns, suggesting a larger steady-state bias.

However, a significant divergence is observed in terms of stability. The RMS offset for phc2sys reaches approximately 420 ns, compared to 157 ns for chrony, indicating substantially higher short-term variability. This is further confirmed by the standard deviation values, where phc2sys (around 417 ns) exhibits nearly five times higher dispersion than chrony (around 88 ns). The most notable difference appears in the extreme values. While chrony maintains bounded behavior within a range of approximately 611 ns peak-to-peak, phc2sys exhibits large excursions, reaching up to +5297 ns, resulting in a peak-to-peak variation exceeding 6 µs. These results indicate the presence of significant outliers or instability events in the phc2sys configuration over longer observation periods.

Overall, while phc2sys provides better mean alignment, its long-term stability under the tested conditions is considerably worse than that of chrony. In contrast, chrony demonstrates more consistent and robust behavior, with tighter offset distribution and significantly reduced sensitivity to transient disturbances. This suggests that chrony’s more conservative control strategy offers improved resilience over extended operation, whereas phc2sys may require additional tuning or filtering to mitigate large timing excursions.

## 4. Multi-Node Synchronization Proof

### 4.1. Inter-Slave Offset Comparison Between Client Nodes

By adding a third RPI5, we will extend our setup in order to show that the scenario established previously is applicable with success for extension to multiple targets. In the multi-node validation setup, one RPI5 acts as the PTP grandmaster, while two RPI5 client nodes operate as independent slaves. Both slaves are synchronized exclusively through PTP and operated without any shared application-level triggers. The implementation is systemd-based.

The correct operation of the PTP synchronization stack was verified against each client target present within the network. Only the relevant command outputs are reported below to demonstrate successful synchronization, while full logs were omitted for clarity. On the master node, both ptp4l and phc2sys services were confirmed to be running. The ptp4l daemon was operating in hardware time stamping mode and assumed the grandmaster role, while phc2sys actively disciplined the system clock using PHC.

#MASTER
/usr/sbin/ptp4l -i eth0 -H -2 -m
/usr/sbin/phc2sys -s /dev/ptp0 -c CLOCK_REALTIME -O 0 -m
#CLIENTS
usr/sbin/ptp4l -i eth0 -H -2 -s -m
/usr/sbin/phc2sys -s /dev/ptp0 -c CLOCK_REALTIME -O 0 -m
The PTP state transitions recorded in the ptp4l log confirm that the local clock was selected as the best master (selected local clock 2ccf67.fffe.f62eb3 as best master) and that the node assumed the grandmaster role (LISTENING to MASTER on ANNOUNCE_RECEIPT_TIMEOUT_EXPIRES, assuming the grand master role).

On the client nodes, the ptp4l daemon successfully detected the master clock and transitioned to the SLAVE state, indicating correct operation of the Best Master Clock Algorithm (BMCA):

new foreign master 2ccf67.fffe.f62eb3-1
selected best master clock 2ccf67.fffe.f62eb3
LISTENING to UNCALIBRATED on RS_SLAVE
master offset 7200016624080 s0 freq   -0 path delay   5080

On the PHC, synchronization was established, the phc2sys service converged and maintained a small offset between the PHC and the system clocks, as shown by the steady state log entries:

CLOCK_REALTIME phc offset -28 s2 freq +43289 delay 37
CLOCK_REALTIME phc offset  44 s2 freq +43353 delay 55

In configuration using chrony for system clock discipline, the chrony daemon was verified to be active and running continuously on all nodes:

systemctl status chrony
Active: active (running)
systemd[1]: Starting chrony.service - chrony, an NTP client/server...
#MASTER: chronyd[6074]: Frequency 40.376 +/- 0.003 ppm read from /var/lib/chrony/chrony.drift
#CLINET1: chronyd[330497]: Frequency 43.521 +/- 0.014 ppm read from /var/lib/chrony/chrony.drift
#CLIENT2: chronyd[329396]: Frequency 44.039 +/- 0.026 ppm read from /var/lib/chrony/chrony.drift

Therefore, we have two scenarios. The first scenario is PTP and phc2sys, and the second scenario is PTP and chrony. Both scenarios have one master target and two client targets. According to the explanation given above, synchronization was successful. Inter-slave phase coherence can be evaluated by comparing the system clock offsets between two independently synchronized client nodes. The same inter-slave alignment and differencing methodology was applied to both chrony- and phc2sys-based configurations. In the phc2sys case, inter-slave coherence was evaluated using PHC–system clock offsets instead of chrony offsets, reflecting the direct clock discipline mechanism.

**Figure 16:**
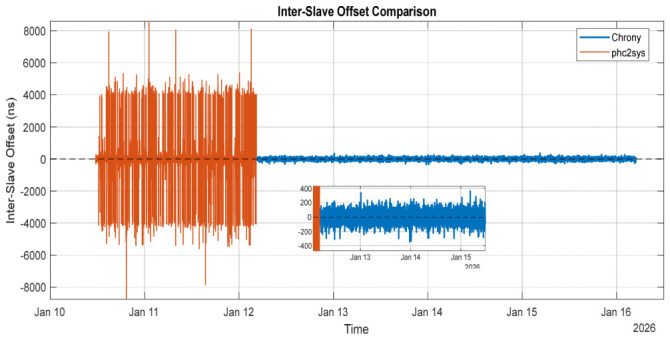
Inter-slave (client nodes only) offset comparison: chrony (blue) and phc2sys (red).

Figure 16 presents the inter-slave differences obtained from long-term (days) monitoring of two PTP-synchronized client nodes disciplined using phc2sys at the beginning of the measuring interval and switching to chrony for the last part of the measuring interval.

For a good interpretation of this figure, inter-slave phase error KPIs can bring more accuracy in the interpretation of the graph, as shown in Table 7. When using chrony, the inter-slave offset exhibited a standard deviation of around 44 ns and a maximum deviation below 400 ns, indicating strong noise filtering and stable long-term behavior. In contrast, phc2sys achieved a similarly unbiased mean offset but exhibited significantly higher short-term variability (σ ≈ 1 µs) and larger transient excursions (up to 8.8 µs), reflecting its aggressive tracking of PHC updates and reduced filtering of network and hardware timestamp noise.

**Figure 17:**
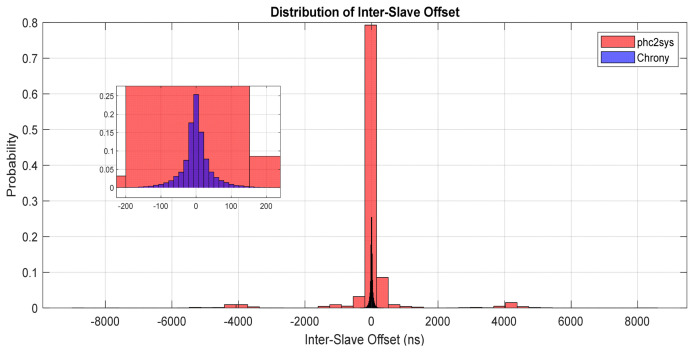
Histogram distribution of inter-slave offset between the two client nodes.

In Figure 17, the histogram represents the probability distribution of the inter-slave system offset, where each bin corresponds to the fraction of samples falling within a given offset range. This normalization enables direct comparison between chrony and phc2sys experiments despite differing run durations. Chrony concentrates most probability mass near zero offset, while phc2sys exhibits heavier tails, indicating increased susceptibility to short-term synchronization noise.

The experimental results highlight distinct and complementary behaviors of chrony and phc2sys when used for disciplining the system clock from a PHC. Chrony demonstrates excellent long-term stability, characterized by strong noise rejection and very tight coherence among slave nodes. While its instantaneous phase lock to the reference is slightly looser, the resulting time evolution is smooth and well controlled.

In contrast, phc2sys achieves a very tight instantaneous phase lock to the PHC, closely tracking the hardware reference. This behavior results in faithful reproduction of hardware- and network-induced noise at the system clock level, leading to increased short-term jitter and occasional larger phase excursions.

These observations indicate that neither approach is universally superior; instead, they are optimized for different performance objectives. Chrony favors stability and coherence in distributed systems, whereas phc2sys prioritizes minimal instantaneous offset to the hardware reference. The choice between the two should, therefore, be guided by the specific accuracy, stability, and noise tolerance requirements of the target application.

### 4.2. Capture of Synchronized Timedate System Signals

Timestamp synchronization nodes are achieved using coordinated systemd service and timer units responsible for RTC-to-system alignment, PHC discipline (via chrony or ph2sys), network-based time convergence using ptp4l, and periodic correction of RTC drift. These mechanisms collectively establish a stable temporal reference across all acquisition nodes, enabling timestamps alignment at microsecond-level precision. To validate the effectiveness of this synchronization framework, two complementary evaluation scenarios are considered. The first scenario is hardware-oriented and involves injecting a common physical trigger signal into all nodes to directly assess timestamp alignment at the GPIO level. The second scenario is software-oriented, where signals are generated independently on each node, software-based only, initiated using triggering conditions, allowing evaluation of timestamp consistency in fully distributed operation.

#### 4.2.1. Multi-Node Hardware Timestamp Synchronization Evaluation

**Figure 18:**
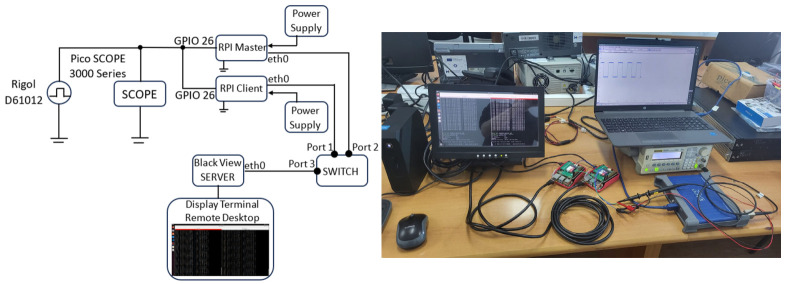
Experimental setup for inter-node timestamp synchronization validation.

The experimental setup used for multi-node timestamp synchronization validation is illustrated in Figure 18. The service file units are already preset, as described in this paper. A periodic reference signal is generated using a Rigol DG1012 function generator and distributed simultaneously to multiple RPI5 nodes via their GPIO inputs (GPIO 26). This common trigger ensures that all nodes receive an identical physical event, enabling direct evaluation of inter-node timestamp consistency. The trigger signal is configured as a 0.1 Hz (10 s period) square wave, allowing repeatable and long-duration evaluation of synchronization stability. The generated signal is monitored using a Pico Scope 3000 Series oscilloscope to verify signal integrity and timing characteristics. Each Raspberry Pi node operates as an independent acquisition unit and is connected to a dedicated Ethernet network through a standard switch. The nodes are powered independently.

The acquisition of synchronization events on each node was implemented using a custom Python-based GPIO monitoring tool. The program detects rising edges on GPIO 26 using a high-resolution monotonic clock (timer.perf_counter_ns()), ensuring nanosecond-level timestamp precision independent of system clock adjustment. To ensure portability across platforms, the implementation supports both the RPi.GPIO library (for Raspberry Pi) and the libgpiod interface for generic Linux-based systems. The polling mode, maximum timing precision at the cost of CPU usage, was selected for experimental validation, as it provides the lowest jitter in Python-based acquisition. Each detected event is timestamped using both monotonic time and a reconstructed wall clock, enabling cross-node comparison.

The recorded timestamps exhibit a highly stable inter-event interval of approximately 10 s, consistent with the configured signal generator period. Small variations observed in the nanosecond domain are attributed to software scheduling latency, GPIO polling granularity, and operating system jitter. A discontinuity between event indices 226 and 227 corresponds to an intentional pause in data acquisition of approximately 2.5 h (see Table 8). This pause does not affect the validity of synchronization analysis, as each measurement window is evaluated independently and later concatenated for statistical assessment.

**Figure 19:**
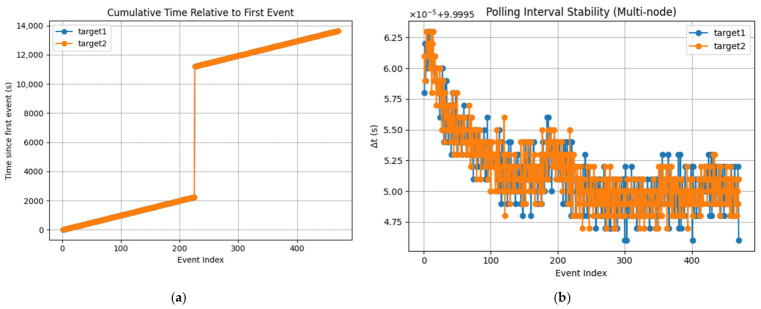
Delta-time stability plot: (**a**) cumulative time/event evolution; (**b**) inter-event interval stability.

Figure 19a presents the cumulative evolution of timestamps relative to the first detected event for each node. The nearly linear trend observed for both targets confirms that the acquisition process is driven by a stable periodic input signal with a nominal period of 10 s (0.1 Hz). The absence of visible divergence between the curves indicates that both nodes maintain a consistent time base over the measurement interval. The slope of the curves reflects the effective sampling period, while the close overlap between nodes demonstrates that long-term drift between the synchronized systems is negligible. A discontinuity in the curve corresponds to the intentional pause between the two acquisition windows. After this pause, the linear trend resumes with the same slope, confirming that synchronization is preserved across restarts and extended idle periods.

Figure 19b illustrates the inter-event interval (Δt) computed as the difference between consecutive timestamps. The results show that the measurement intervals are tightly clustered around the expected value of 10 s, confirming the stability of the signal generation and acquisition process. Small fluctuations in the order of microseconds are observed and can be attributed to software-related factors such as operating system scheduling, GPIO polling latency, and Python execution overhead.

Despite these effects, the variation remains minimal and does not impact the overall temporal consistency of the measurements. The continuity of Δt values within each acquisition window demonstrates stable operation, while the absence of abnormal pikes confirms that no events were missed during acquisition. This validates the robustness of the polling-based detection approach used in this study.

**Figure 20:**
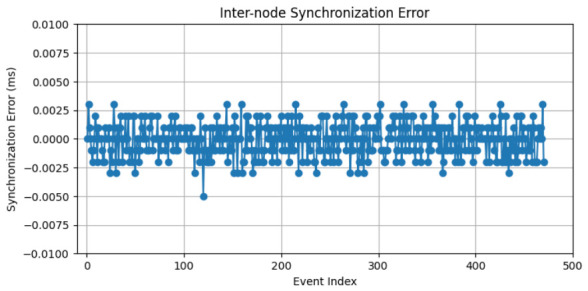
Inter-node synchronization error.

In Figure 20, the synchronization error between nodes is shown, computed as the difference between corresponding timestamps acquired on the master and client systems. The results show that the synchronization error remains bounded within a narrow range, typically at the microsecond level. This level of agreement demonstrates the effectiveness of the underlying time synchronization infrastructure, including RTC alignment, PTP-based clock discipline, and system-level time management. The absence of long-term drift in the error signal confirms that both nodes remain phase-aligned over extended operation periods.

#### 4.2.2. Multi-Node Software Timestamp Synchronization Evaluation

To complement the hardware acquisition experiment described in Section 4.2.1, a software emulation scenario was also evaluated. In this scenario, each RPI5 node generates synthetic three-phase voltage and current waveforms in software using Python 3.12.3-based scientific libraries [64], allowing a controlled assessment of timestamp alignment independently of electrical front-end and signal transmission effects. The primary objective is to demonstrate and validate the synchronization of signals collected from multiple timestamped-synchronized nodes in real time. Each node acts as an RPI5 measurement target, generating synthetic three-phase voltage and current waveforms at 50 Hz, with nominal values of 230 V and 5 A, respectively. To approximate realistic operating conditions, the voltage amplitudes are randomly perturbed within ±3%, while current amplitudes are varied within ±20%, reflecting typical load fluctuations and measurement noise. Each waveform spans a duration of approximately 0.2 s and is discretized into 400 samples over two periods of the sinusoidal signal. Signal generation is triggered by a central control script that runs concurrently across multiple nodes. Each node continuously produces signals for a defined duration and stores them as timestamped CSV files, organized by target (e.g., “target1”, “target2”, “target3”), ensuring a clear temporal ordering of measurements across all nodes.

[target3] Fetched target3_20260124T120951.csv
[target2] Fetched target2_20260124T120951.csv
[target1] Fetched target1_20260124T120951.csv
[target2] Fetched target2_20260124T122113.csv
[target3] Fetched target3_20260124T122113.csv
[target1] Fetched target1_20260124T122113.csv
…
[target1] Fetched target1_20260207T105904.csv
[target3] Fetched target3_20260207T105904.csv
[target2] Fetched target2_20260207T105904.csv
[target2] Fetched target2_20260207T110019.csv
[target1] Fetched target1_20260207T110019.csv
[target3] Fetched target3_20260207T110019.csv
…
[target1] Fetched target1_20260214T095214.csv
[target3] Fetched target3_20260214T095214.csv
[target2] Fetched target2_20260214T095214.csv

In a realistic deployment, each target could correspond to a RPI5 collecting data. The central system (server target) automatically fetches newly created CSV files from each node in near real time, storing them in a local directory. This process guarantees that signals from all nodes are synchronized and available for simultaneous analysis, enabling verification of temporal alignment even when data are generated independently.

It is important to clarify the role of clock jitter in the software emulation scenario. In this experiment, no external analog signal is acquired; instead, each node generates synthetic waveforms and timestamps them using the system clock (CLOCK_REALTIME), which is synchronized through the PTP/chrony framework described in Section 3.

Under these conditions, the timestamps reflect the accuracy and jitter characteristics of the disciplined system clock. They are not affected by ADC sampling jitter, GPIO interrupt latency, or analog front-end noise, as these hardware components are not exercised.

Therefore, the inter-node timestamp agreement observed in this setup represents the synchronization performance of the software timekeeping layer in isolation. This result can be regarded as an upper bound on synchronization accuracy, corresponding to ideal acquisition conditions with negligible sampling-related jitter.

The hardware experiment presented in Section 4.2.1 provides a complementary assessment under practical operating conditions, where GPIO interrupt latency and kernel scheduling variability introduce additional timing uncertainty. As such, it yields a more conservative estimate of achievable synchronization performance.

Taken together, these two experiments establish a bounded range for inter-node synchronization accuracy in distributed sensing systems.

The collected data are visualized through a web-based dashboard implemented in Dash and Plotly, structured into two main tabs with distinct functionalities. The Dynamic RMS Plotting Tab supports real-time monitoring of synchronized measurements across nodes. When the Start Measurement checkbox is enabled, a control script is triggered, receiving two key arguments: --duration, which defines the total acquisition time in seconds, and --sleep, which sets the interval between consecutive signal recordings. Both parameters can be specified directly from the dashboard interface, being a user decision. The trigger script launches a measurement agent script on each node, generating three-phase voltage and current signals at a nominal frequency of 50 Hz, with random amplitude variations applied to simulate realistic fluctuations. These signals are collected into timestamped CSV files in a dedicated data directory at a cadence corresponding to the specified sleep interval, continuing until the data acquisition time is reached.

**Figure 21:**
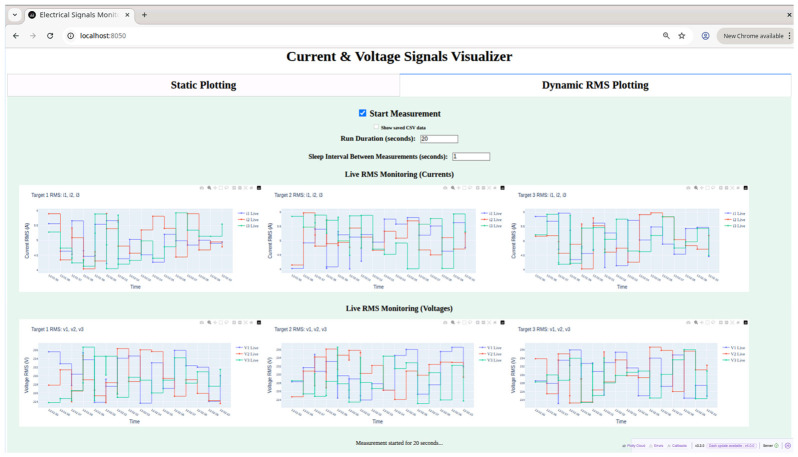
Dynamic RMS Plotting Tab from the deployed Dash Plotly Dashboard.

Meanwhile, the dashboard runs parallel background threads that continuously poll the nodes for new CSV files and retrieve them as soon as they become available (see Figure 21). Once the data is collected, the dashboard automatically computes RMS values for each phase and each node and updates the plots at regular intervals. The user can view the RMS evolution for all nodes simultaneously, which allows direct evaluation of both amplitude variability and temporal alignment between nodes.

The design of this tab ensures that acquisition is presented in an aggregated, synchronized view, clearly highlighting the coordination between distributed measurements. Additionally, previously saved CSV data can be loaded for comparison with live data, enabling offline verification of signal synchronization and amplitude consistency.

The Static Plotting Tab allows users to explore previously collected signals stored in timestamped CSV files. Users can filter the dataset by selecting a desired time range and choosing one or multiple files for analysis.

For single file selection, if only one CSV file is selected, the dashboard presents dropdown menus containing all the signals from that file, allowing users to plot any combination of voltage and current traces, or all signals simultaneously.

**Figure 22:**
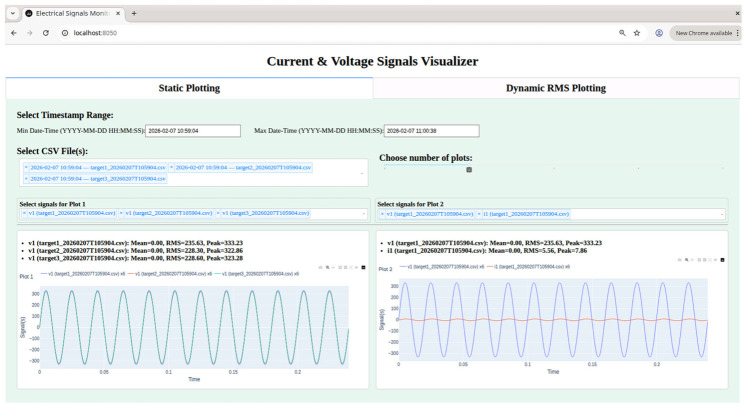
Static Plotting Tab from the deployed Dash Plotly Dashboard.

Multiple file selections (what can be seen also from Figure 22) is when multiple CSV files are selected, the dashboard aggregates all signals across the chosen files into the dropdown menus. This gives users the flexibility to combine and plot multiple signals, whether they are voltages, currents, or a combination of both, from the same nodes or across different nodes.

Once the signals are selected, interactive plots display waveform evolution over time. Alongside each plot, key metrics such as mean, RMS, and peak values are automatically computed as shown, allowing rapid assessment of signal amplitude and characteristics. By visualizing signals from multiple nodes together, the tab highlights temporal alignment, making plots displayed simultaneously to facilitate direct comparison across phases, targets, or measurement types.

The Static Plotting Tab provides a flexible environment for offline analysis, supporting the verification of signal characteristics, amplitude variability, and inter-node temporal coordination. By presenting the data in a structured, interactive, and visually coherent manner, this tab complements the real-time monitoring features of the Dynamic RMS Plotting Tab, offering a comprehensive toolset for both historical and live evaluation of synchronized signals.

Continuous operation over a period exceeding a week, as shown by the timestamps listed in the CSV names above, demonstrated that inter-node timestamps remained consistently aligned to a common master reference, with no measurable long-term divergence. The agreement observed in both real-time and static signal representation confirms stable synchronization and verifies correct end-to-end propagation of hardware-level time discipline through the operating system to the application layer.

## 5. Discussion

The experimental results demonstrate that high-precision, statistically bounded distributed synchronization can be achieved entirely offline using commodity embedded hardware, provided that hardware timestamping, persistent time retention, driver-level IEEE 1588 enablement, and operating system-managed convergence control are treated as integrated architectural components rather than independent configuration choices. Sustained microsecond-level clock offset and bounded dispersion observed over multi-day operation confirm the feasibility of Ethernet-layer hardware timestamping combined with PHC discipline, consistent with industrial evaluations of IEEE 1588 synchronization performance [27,61,62].

### 5.1. Advantages of Wired Ethernet over Wireless Synchronization

Although wireless synchronization technologies are widely adopted in Internet of Things (IoT) and sensor network applications [6,9,15,16,35,69,70,71], their suitability for deterministic nanosecond-level alignment remains limited. Wireless media inherently introduce stochastic latency components arising from channel contention, retransmission mechanisms, interference, and MAC-layer scheduling variability. Secure NTP-based and delay-estimation approaches further illustrate the sensitivity of packet-based time transfer to asymmetry and perturbations [12,13,43,72,73,74]. In contrast, wired Ethernet provides a full-duplex, deterministic physical medium that supports hardware timestamping directly at the MAC/PHY layer, as standardized by IEEE 1588-2019 [61]. Industrial automation studies and Time-Sensitive Networking (TSN) implementations demonstrate that Ethernet-based hardware timestamping can achieve sustained microsecond-bounded synchronization under realistic operational loads [23,24,25,27]. Therefore, Ethernet was selected not merely for implementation convenience but because it minimizes stochastic latency components and enables hardware-layer timestamp acquisition essential for statistically bounded offline synchronization.

### 5.2. Role of the Raspberry Pi 5 Platform

A critical enabler of the observed performance is the evolution of the Raspberry Pi platform itself [35,59]. Compared to earlier Raspberry Pi 3 and Raspberry Pi 4 generations, Raspberry Pi 5 introduces significant improvements in processing capability, I/O throughput, and peripheral architecture [30,35,56,59,60]. Of particular importance is the availability of a native battery-backed real-time clock (RTC), eliminating the need for external RTC modules previously required for deterministic restart after power interruption [30,48]. Earlier platform generations exhibited limited or inconsistent hardware timestamping support at the Ethernet controller level [2,47], whereas the improved Ethernet subsystem in RPI5 enables tighter convergence control, deterministic restart behavior, and sustained clock discipline without reliance on external time references.

The maturity of Ethernet controller drivers and Linux networking subsystem further contributes to synchronization accuracy. Prior investigations have shown that interrupt moderation, buffering mechanisms, and kernel-level scheduling behavior significantly influence timestamp determinism in embedded systems [29,47,63]. While some implementations rely on external Intel network adapters connected to Raspberry Pi platforms for PTP validation [33], the present architecture achieves stable hardware-level timestamping directly on the integrated Cadence MACB/GEM Ethernet controller using standard ptp4l utilities. This reduces architectural complexity, lowers hardware dependencies, and improves portability toward future single-board computer generations as native MAC/PHY timestamping support continues to mature.

### 5.3. Decoupling of Synchronization Evaluation from Payload Transmission

An important distinction concerns payload handling. Approaches that evaluate synchronization exclusively through continuous PTP packet transmission must account for packet scheduling, queueing, and buffering variability, which can introduce measurable clock perturbations or apparent measurement artifacts [17,63,64]. In the proposed framework, synchronization evaluation is decoupled from continuous Ethernet payload transmission. Instead, both hardware-triggered GPIO events (Section 4.2.1) and emulated sensor data (Section 4.2.2) are timestamped using the disciplined system clock, which inherits precision from the PHC. Because synchronization metrics are not derived from transport-level PTP packet exchange behavior, packet-induced latency artifacts do not bias the offset measurements. This design choice enables a cleaner separation between the synchronization infrastructure and the data acquisition layer, facilitating independent characterization of each subsystem.

### 5.4. Chrony Versus phc2sys: Comparative Performance

The quantitative comparison between chrony-based and phc2sys-based system clock disciplines reveals distinct and complementary performance characteristics, each suited to different operational priorities. For PHC-to-system clock discipline under identical conditions over a 24 h observation window, chrony achieved a mean offset of approximately +129 ns with a standard deviation of 88 ns and a peak-to-peak variation of 611 ns (−201 ns to +410 ns), whereas phc2sys yielded a lower mean offset of approximately +51 ns but exhibited substantially higher variability, with a standard deviation of 417 ns and a peak-to-peak deviation of 6147 ns (−850 ns to +5297 ns).

These results highlight a fundamental trade-off. The phc2sys servo applies rapid, direct corrections that track the PHC with minimal latency, achieving tighter mean phase alignment but at the cost of faithfully reproducing hardware- and network-induced noise at the system clock level. Chrony, in contrast, employs a more conservative filtering strategy that suppresses short-term jitter and limits large transient excursions, resulting in a modestly higher steady-state bias but significantly improved stability over extended operation. This behavior is consistent with known differences between hardware-aligned and filtering-driven clock discipline mechanisms reported in the literature [27,61].

From the perspective of distributed electrical signal monitoring, the tighter dispersion and bounded peak-to-peak behavior of the chrony configuration are particularly advantageous. Phase estimation accuracy and harmonic analysis in 50 Hz systems are more sensitive to sustained random jitter than to a small, stable mean offset, which can be compensated through calibration. The inter-slave coherence results presented in Section 4.1 further reinforce this conclusion. With chrony, the inter-slave offset exhibited a standard deviation of approximately 44 ns and a maximum absolute deviation below 400 ns, compared to approximately 1005 ns and 8760 ns, respectively, with phc2sys. Accordingly, chrony-based discipline is recommended as the default configuration for applications that prioritize long-term stability and inter-node coherence, while phc2sys may be preferred in scenarios demanding minimal instantaneous offset to the hardware reference, provided that additional noise filtering is applied.

### 5.5. Validation Methodology and Limitations

It is important to acknowledge a limitation of the validation methodology. In the hardware experiment described in Section 4.2.1, the reference signal generated by the function generator provides a common physical trigger simultaneously distributed to all nodes, enabling direct evaluation of inter-node timestamp consistency independently of the synchronization toolchain. The oscilloscope confirms signal integrity and periodicity. However, the absolute accuracy of the system clocks relative to an external ground-truth reference, such as a GPS-disciplined or atomic clock primary standard, has not been independently verified. The synchronization metrics (ptp4l master offset, phc2sys offset, chrony tracking) are produced by the same toolchain that implements the discipline; they are, therefore, self-consistent but not externally calibrated. The inter-node timestamp differences derived in Section 4.2.1 from the common physical trigger constitute a more independent measurement, since they compare events recorded on different synchronized clocks against a shared physical reference. The nanosecond-to-microsecond agreement observed there supports the validity of the claimed synchronization performance; nevertheless, future work should include calibration against a GPS-disciplined or IEEE 1588 grandmaster of known traceability in order to provide a fully independent absolute accuracy characterization.

Although the graphical results presented in Section 3 illustrate representative day-scale intervals, extended multi-week experiments revealed no observable cumulative drift accumulation, consistent with bounded synchronization behavior reported in industrial PTP deployments [25,27]. Further environmental stress testing, including systematic characterization of thermal variation effects on crystal oscillator frequency drift, could provide additional quantitative insight into long-term robustness limits in outdoor or thermally uncontrolled deployments.

### 5.6. Interaction Between Synchronization and Data Acquisition

The interaction between synchronization and data acquisition subsystems represents another practical consideration. In realistic sensing scenarios, GPIO-triggered acquisition and I^2^C-based sensor polling introduced interrupt load, bus arbitration delays, and kernel scheduling variability [43]. If not carefully managed, such factors may influence timestamp determinism independently of the underlying clock discipline accuracy. Extending the framework toward real hardware acquisition using LEM transducers, voltage adaptation circuits and GPIO-triggered sampling constitute a natural progression of this work. Under those conditions, long-term phase coherence of 50 Hz signals and cumulative phase error over multi-day operation become critical performance indicators, particularly for grid-connected measurement applications.

### 5.7. Scalability and Future Directions

The proposed architecture establishes a scalable foundation for advanced power quality analysis integrated directly within the visualization environment. Because all distributed nodes maintain statistically bounded microsecond-level timestamp coherence, higher-order metrics such as Fast Fourier Transform (FFT)-based spectral analysis, Total Harmonic Distortion (THD), and power factor computation can be computed reliably across distributed acquisition channels. Precise temporal alignment [6] ensures stable phase relationships between voltage and current signals over extended operation, enabling accurate harmonic decomposition and long-term power factor analysis.

Several limitations of the present validation warrant acknowledgement. First, the experimental setup comprises three RPI5 nodes of identical hardware specification operating in a controlled laboratory environment with stable ambient temperature. The synchronization performance of the proposed architecture under heterogeneous hardware, for instance, combining RPI5 nodes with devices whose Ethernet controllers provide only software-level PTP timestamping, has not been evaluated. In such mixed environments, the grandmaster-to-slave offset contribution from a software-timestamped client would be expected to degrade to the tens-of-microseconds range, consistent with known software timestamping limitations [27,61]. Second, network scalability beyond three nodes was not experimentally evaluated. Scaling a wired PTP network introduces path delay asymmetries and switch queuing effects that may affect synchronization accuracy as node count increases. Industrial evaluations suggest that well-designed IEEE 1588 networks can sustain sub-microsecond accuracy across tens of nodes [24,27], but this has not been verified in the present deployment. Third, thermal variation was not systematically characterized. Crystal oscillator frequency drift is temperature-dependent, and in outdoor or thermally uncontrolled deployments, the effective RTC and PHC drift rates may differ from the laboratory values reported here. Extending the experimental characterization to cover heterogeneous hardware, larger node counts, and thermal excursions constitutes a natural direction for future work.

## 6. Conclusions

The paper presents the design, system-level implementation, and long-duration experimental validation of a fully autonomous offline time synchronization architecture for distributed electrical signal monitoring systems built on RPI5 platforms interconnected through a private Ethernet network. The proposed framework was developed as an integrated operating system-level solution, combining hardware timestamping, layered clock discipline mechanisms, persistent time retention, and deterministic service orchestration into a coherent architectural layer that operates without any external time reference.

A distinguished aspect of this work is the exploitation and extension of new hardware capabilities introduced in RPI5. In particular, the integration of the J5 battery-backed real-time clock enables deterministic restoration of valid system time after power interruption, eliminating dependency on external references during boot. Furthermore, enabling and establishing PTP support within the Cadence MACB/GEM driver at the Ethernet controller level ensures that hardware timestamping occurs directly at the network interface, reducing software-induced jitter and enabling precise PHC discipline across distributed nodes. When combined with system-managed startup sequencing, the architecture guarantees controlled synchronization convergence, ordered activation of timing services, and predictable recovery behavior following both controlled and uncontrolled shutdown events, transforming synchronization from an isolated tool configuration into an embedded architectural function of the operating system.

The framework supports both chrony-based PHC-to-system discipline and direct phc2sys synchronization, enabling comparative analysis of filtering-driven versus hardware-aligned clock strategies. The quantitative results obtained from continuous multi-day experimental campaigns demonstrate sustained microsecond-level mean offsets, with clearly distinct stability characteristics between the two discipline mechanisms. For chrony-based discipline, a mean PHC-to-system offset of approximately +129 ns was achieved, with a standard deviation of 88 ns and a bounded peak-to-peak variation of 611 ns. The phc2sys configuration yielded a lower mean offset of approximately +51 ns, indicating improved centering of the system clock relative to the PHC, but it exhibited significantly higher variability with a standard deviation of 417 ns and a peak-to-peak deviation exceeding 6 µs. These results indicate that, while phc2sys minimizes steady-state offset, it is more susceptible to transient excursions and short-term instability over extended observation intervals. Chrony, by comparison, demonstrates more consistent bounded dispersion, effectively limiting jitter and suppressing large deviations through its hybrid frequency-filtering control strategy. The inter-slave offset measurements further confirm this conclusion: under chrony-based discipline, the standard deviation of inter-node clock differences was approximately 44 ns with a maximum absolute deviation below 400 ns compared to approximately 1005 ns and 8760 ns under phc2sys.

No progressive drift accumulation was observed throughout the extended evaluation window, confirming effective long-term frequency discipline for both approaches. Controlled power interruption experiments further validate deterministic recovery enabled by RTC persistence, MACB-based hardware timestamping continuity, and systemd- coordinated initialization. The proposed periodic system-to-RTC correction mechanism, implemented as an hourly systemd timer–service pair, was shown to suppress long-term RTC drift and ensure reliable boot time clock restoration across repeated power cycles.

End-to-end propagation of hardware-level synchronization to the application layer was validated through two complementary experiments. In the hardware experiment (Section 4.2.1), a common periodic physical signal was simultaneously acquired by all nodes via GPIO inputs, and the inter-node timestamp deviations were evaluated against the shared reference, confirming microsecond-level alignment under real acquisition conditions. In the software experiment (Section 4.2.2), synthetic three-phase voltage and current waveforms were generated and timestamped independently on each node, with temporal alignment verified through dynamic interactive visualization implemented in Dash and Plotly. The use of responsive callback mechanisms allowed precise alignment analysis, while seamless integration with NumPy and SciPy facilitated reproducible post-processing and rapid methodological validation. Continuous monitoring for over one week confirmed that timestamps remained consistently aligned with no measurable long-term divergence, validating correct end-to-end propagation of hardware-level synchronization discipline through the operating system to the application layer.

The results confirm that precise distributed synchronization is achievable using low-cost embedded platforms when synchronization is designed as a system-level architectural layer rather than as an application-level feature. The proposed architecture provides a reproducible and extensible foundation for offline distributed monitoring in scenarios where conventional network-dependent synchronization methods are non-viable. Future work will focus on extending the validation to heterogeneous hardware configurations, larger node counts, and thermally uncontrolled environments, as well as integrating the framework with real hardware acquisition front-ends employing LEM transducers and GPIO-triggered analog-to-digital conversion for long-term power quality analysis in grid-connected applications.

## Figures and Tables

**Table 1:** Timedatectl reported dates after more than 48 h.

Target 1		Target 2
Start measuring time dates		
Local time: Universal time: RTC time:	Fri 2025-12-12 16:18:23 UTC Fri 2025-12-12 16:18:23 UTC Fri 2025-12-12 16:18:23	Fri 2025-12-12 16:18:23 UTC Fri 2025-12-12 16:18:23 UTC Fri 2025-12-12 16:18:23
After more than 48 h of recording time dates		
Local time: Universal time: RTC time:	Mon 2025-12-15 08:09:24 UTC Mon 2025-12-15 08:09:24 UTC Mon 2025-12-15 08:09:20	Mon 2025-12-15 08:09:26 UTC Mon 2025-12-15 08:09:26 UTC Mon 2025-12-15 08:09:19

**Table 2:** Chrony config files for the master and client settings.

Target 1	Target 2
# Chrony MASTER (no internet) # Do NOT use internet pools # Act as local time source local stratum 8 allow 192.168.0.0/24 # Keep RTC aligned rtcsync # Allow initial correction makestep 1 3 # Drift tracking driftfile /var/lib/chrony/chrony.drift logdir /var/log/chrony	# Chrony CLIENT (private network) # Use master as time source server <RPI5_masterIP> iburst # Keep RTC aligned rtcsync # Allow initial correction makestep 1 3 # Drift tracking driftfile /var/lib/chrony/chrony.drift logdir /var/log/chrony

**Table 3:** ptp4l systemd services for master and client nodes.

Target 1	Target 2
#MASTER /etc/systemd/system/ptp4l-master.service [Unit] Description=PTP4L Grandmaster (eth0) After=rtc-to-system.service Wants=rtc-to-system.service [Service] Type=simple ExecStart=/usr/sbin/ptp4l -i eth0 -H -2 -m Restart=always RestartSec=2 # Logging (compatible with logrotate) StandardOutput=append:%L/ptp4l.log StandardError=append:%L/ptp4l.log [Install] WantedBy=multi-user.target	#CLIENT /etc/systemd/system/ptp4l-client.service [Unit] Description=PTP4L Slave (eth0) After=rtc-to-system.service Wants=rtc-to-system.service [Service] Type=simple ExecStart=/usr/sbin/ptp4l -i eth0 -H -2 -s -m Restart=always RestartSec=2 # Automatic log directory creation StandardOutput=append:%L/ptp4l.log StandardError=append:%L/ptp4l.log [Install] WantedBy=multi-user.target

**Table 4:** Chrony config file settings for master and client targets.

Target 1	Target 2
# MASTER # Chrony MASTER (no internet) # Use PHC as the primary time source refclock PHC /dev/ptp0 poll 0 dpoll -2 # Act as local time source if needed local stratum 8 allow 192.168.0.0/24 # Keep RTC aligned rtcsync # Allow initial correction makestep 1 3 # Drift tracking driftfile /var/lib/chrony/chrony.drift logdir /var/log/chrony	# CLIENT # Chrony CLIENT (private network) # Chrony using PTP Hardware Clock (PHC) # Use PHC as the primary time source refclock PHC /dev/ptp0 poll 0 dpoll -2 offset 0 # Keep RTC aligned rtcsync # Allow intial correction makestep 1 3 # Drift tracking driftfile /var/lib/chrony/chrony.drift logdir /var/log/chrony

**Table 5:** phc2sys config file settings for master and client targets.

Target 1	Target 2
# MASTER /etc/systemd/system/phc2sys-master.service: [Unit] Description=PHC to System Clock (PTP Master) After=ptp4l-master.service Requires=ptp4l-master.service [Service] Type=simple ExecStart=/usr/sbin/phc2sys \ -s /dev/ptp0 \ -c CLOCK_REALTIME \ -O 0 \ -m Restart=always RestartSec=2 # Automatic log directory creation StandardOutput=append:%L/phc2sys.log StandardError=append:%L/phc2sys.log [Install] WantedBy=multi-user.target	# CLIENT /etc/systemd/system/phc2sys-client.service: [Unit] Description=PHC to System Clock (PTP Client) After=ptp4l-client.service Requires=ptp4l-client.service [Service] Type=simple ExecStart=/usr/sbin/phc2sys \ -s /dev/ptp0 \ -c CLOCK_REALTIME \ -O 0 \ -m Restart=always RestartSec=2 # Automatic log directory creation StandardOutput=append:%L/phc2sys.log StandardError=append:%L/phc2sys.log [Install] WantedBy=multi-user.target

**Table 6:** Quantitative results.

Metric	chrony (PHC → System)	phc2sys (PHC → System)
Mean offset	+129.45 ns	+50.63 ns
RMS offset	156.70 ns	420.37 ns
Standard deviation	88.30 ns	417.31 ns
Minimum/maximum	−201 ns/+410 ns	−850 ns/+5297 ns
Peak-to-peak	611 ns	6147 ns

**Table 7:** Chrony config files settings for master and client nodes.

Inter-Slave PHC–SYS Offset KPIs	
chrony	phc2sys
Mean Δchrony offset: −0.06 ns Std Δchrony offset: 43.98 ns Max |Δchrony|: 364.00 ns	Mean ΔPHC–SYS offset: 8.32 ns Std ΔPHC–SYS offset: 1005.02 ns Max |ΔPHC–SYS|: 8760.00 ns

**Table 8:** Timestamp events on both master and client nodes.

Target 1—Master	Target 2—Client
Begin—first window events index and timestamps of microsecond-level order	
[# 1] 2026-03-27 06:13:57.063208 | mono=2_588_949_333_209 ns [# 2] 2026-03-27 06:14:07.062766 | mono=2_598_948_890_686 ns [# 3] 2026-03-27 06:14:17.062328 | mono=2_608_948_452_926 ns [# 4] 2026-03-27 06:14:27.061887 | mono=2_618_948_012_109 ns	[# 1] 2026-03-27 06:13:57.063205 | mono=2_659_873_353_962 ns [# 2] 2026-03-27 06:14:07.062766 | mono=2_669_872_915_213 ns [# 3] 2026-03-27 06:14:17.062325 | mono=2_679_872_474_142 ns [# 4] 2026-03-27 06:14:27.061886 | mono=2_689_872_034_951 ns
Between—windows—events index and timestamps of microsecond-level order	
[# 224] 2026-03-27 06:51:06.963686 | mono=4_818_849_811_088 ns [# 225] 2026-03-27 06:51:16.963238 | mono=4_828_849_363_010 ns [# 226] 2026-03-27 06:51:26.962790 | mono=4_838_848_914_753 ns [# 227] 2026-03-27 09:20:36.560471 |mono=13_788_446_595_836 ns [# 228] 2026-03-27 09:20:46.560023 |mono=13_798_446_147_434 ns [# 229] 2026-03-27 09:20:56.559571 |mono=13_808_445_695_865 ns	[# 224] 2026-03-27 06:51:06.963685 | mono=4_889_773_833_849 ns [# 225] 2026-03-27 06:51:16.963238 | mono=4_899_773_386_537 ns [# 226] 2026-03-27 06:51:26.962790 | mono=4_909_772_938_937 ns [# 227] 2026-03-27 09:20:36.560470|mono=13_859_370_618_499 ns [# 228] 2026-03-27 09:20:46.560022|mono=13_869_370_170_214 ns [# 229] 2026-03-27 09:20:56.559573|mono=13_879_369_721_404 ns
End—second window events index and timestamps of microsecond-level order	
[# 469] 2026-03-27 10:00:56.451506 |mono=16_208_337_630_749 ns [# 470] 2026-03-27 10:01:06.451055 |mono=16_218_337_179_418 ns [# 471] 2026-03-27 10:01:16.450607 |mono=16_228_336_731_392 ns [# 472] 2026-03-27 10:01:26.450153 |mono=16_238_336_277_890 ns	[# 469] 2026-03-27 10:00:56.451505|mono=16_279_261_653_997 ns [# 470] 2026-03-27 10:01:06.451055|mono=16_289_261_203_798 ns [# 471] 2026-03-27 10:01:16.450604|mono=16_299_260_752_872 ns [# 472] 2026-03-27 10:01:26.450155|mono=16_309_260_303_102 ns

## Data Availability

Data are contained within the article.
